# Comprehensive Medication Management Services with a Holistic Point of View, a Scoping Review

**DOI:** 10.3390/pharmacy11010037

**Published:** 2023-02-16

**Authors:** Evelyn I. Rojas, Niurka M. Dupotey, Hans De Loof

**Affiliations:** 1 Departamento de Farmacia, Facultad de Ciencias Naturales y Exactas, Universidad de Oriente, Patricio Lumumba Avenue, Altos de Quintero, Santiago de Cuba City 90500, Cuba; 2 Laboratory of Physiopharmacology, University of Antwerp, Universiteitsplein 1, B-2610 Antwerp, Belgium

**Keywords:** comprehensive medication therapy management, comprehensive medication management, scoping review, implementation, holistic approach

## Abstract

Implementing Comprehensive Medication Management (CMM) services uncovered the importance of the totality of the patient’s perspective in this process. The holistic approach takes into account the physical, mental and emotional well-being of individuals, as well as their socioeconomic circumstances. The aim of this study was to characterize the scientific evidence associated with CMM services that included this holistic approach. A scoping review was conducted based on Arksey and O’Malley’s method. Searches were performed in Google Scholar for papers published between 2010 and 2020 in English, Spanish and Portuguese. Study design, health contexts, sample of patients, results obtained, barriers and facilitators, and the integration of a holistic approach were determined. Two hundred and eighteen papers were evaluated, most of which focused on the implementation of this service through prospective observational studies. A minority of studies reported on a holistic approach, a smaller number examined the effect of social determinants of health, the patient’s medication experiences and the pharmacotherapy outcomes from the patient’s perspective. Despite the progress achieved, most of the referents do not yet reflect a broader view of the patient’s life situation and its relationship to pharmacotherapy and the ways in which the pharmacist implements holistic elements to solve or prevent drug-related problems.

## 1. Introduction

In 2017, the World Health Organization (WHO) launched the third global patient safety challenge with the goal of reducing avoidable medication-related harm by 50% over the next five years [1]. A drug-related problem (DRP) is an event or circumstance involving drug therapy that actually or potentially interferes with desired health outcomes [2]. The clinical services aimed at the prevention and resolution of these DRP have become part of pharmaceutical care as defined by Pharmaceutical Care Network Europe Association (PCNE) and include pharmacotherapy follow-up, patient education, pharmacovigilance and drug information [3,4]. Dupotey et al. [5], however, describe the limited availability of these services in health systems. The wide variety of services implemented can result in fragmented patient care. In response to this, comprehensive medication management (CMM) was designed as an integrated way of undertaking pharmaceutical care services [5,6,7].

CMM is defined as the standard of care that ensures each patient’s medications (whether they are prescription, nonprescription, alternative, traditional, vitamins or nutritional supplements) are individually assessed to determine that each medication is appropriate for the patient, effective for the medical condition, safe given the comorbidities and other prescribed medications, and able to be taken by the patient as intended [6,8]. CMM is more comprehensive than medication therapy management (MTM) in that CMM evaluates all medications and all medical conditions, requires a collaborative practice agreement and includes follow-up care to ensure resolution of medication-related problems and attainment of treatment goals. MTM has a tendency to be less integrated with medical practices and with limited clinical information [9].

The implementation of CMM has spread to a large number of health institutions in the United States [10,11], in Canada [12] and Spain [13,14]. Within Latin America, Brazil [15,16] stands out for its early CMM implementation. In several Europe countries, uptake is much lower for numerous reasons [17]. 

There is a growing consensus that healthcare should take into account the physical, mental and emotional well-being of individuals, as well as their socioeconomic circumstances [18]. Incorporation of patients’ subjective experiences [19] and the social determinants of health (SDOH) [20] is therefore needed in what we call a holistic approach to pharmaceutical care. Both additional elements confer special usefulness in the identification, prevention and resolution of DRPs [21,22,23].

Despite the achieved progress, there are numerous barriers that have hindered the development of these services. These include, among others, the lack of resources, the deficit in clinical competence of pharmacists and the limited contact between pharmacist and physicians [24]. Various methodological designs are presented in the studies to demonstrate the outcomes of CMM service in the patient, showing in each of them their strengths and limitations [25,26,27].

For this reason, the analysis of the literature about the implementation of these services has been useful to characterize the development of clinical pharmacy. The aim of this review is to characterize the scientific evidence associated with the development of CMM services and the state of the art of the holistic approach in its implementation.

Previous examination of the literature about the implementation of these services has already been useful to inform further improvements in clinical pharmacy services. The scoping reviews have been conducted on the implementation of medication review services in community pharmacies [28], medicine use review in the United Kingdom [29], medication reconciliation at patient discharge [30], use and impact of telehealth medication reviews [31] and identification of strategies and interventions improving interprofessional collaboration and integration in primary care [32]. This review aims to identify the scientific evidence related to a holistic approach, as described above, without limiting it to a particular healthcare setting. Besides searching the English language scientific literature, we chose to additionally examine the references in Spanish and Portuguese about the CMM services. Our primary aim, in parallel to previous reviews, was to inform and guide the implementation of improved patient-centered CMM services. Secondary objectives were to (1) characterize the scope and nature of publications referring to CMM services; (2) identify the theoretical frameworks used in the process of patient care process; (3) identify the health contexts in which the CMM service was developed and the populations that benefited; (4) identify the main pharmacist intervention carried out and the results measured; (5) identify the main barriers or facilitators revealed by authors; (6) analyze the application of the integration of the holistic approach in the patient care process.

## 2. Materials and Methods

We followed the Arksey and O’Malley five-stage methodology [33] and Preferred Reporting Items extension for Scoping Reviews (PRISMA-ScR) checklist during the execution of this scoping review [34,35].

### 2.1. Search Strategy and Inclusion Criteria 

To provide a wide source of information, including the gray literature, the search was performed in Google Scholar using the Publish or Perish software [36]. The first search was performed in May 2020 and a second search was carried out in November 2021. The query was performed with the following search terms: “Gestión Integral de la Farmacoterapia”, “Gerenciamento da Terapia Medicamentosa” and “Comprehensive Medication Therapy Management”. For the inclusion of papers, the following criteria were used: (a) related to the theoretical framework of the CMM service or its implementation; (b) written in English, Spanish and Portuguese; (c) insertion of gray literature in addition to journal articles and (d) published from 2010 to 2020. We restricted the search to recent developments in view of the evolving nature of the services under study. We did not use other databases because of resource limitations. 

### 2.2. Selection of Information Sources and Data Extraction

After the elimination of duplicates, the titles were read, followed by the abstract, in order to evaluate their inclusion by the first author (EI.R). In the final reading, papers in other languages (not in Spanish, Portuguese and English) or not related to the CMM service were eliminated by consensus with the second author (NMD). Data extraction was performed on the suitable articles in Microsoft Excel. The extracted information was organized as follows: (a) general characteristics of the published work (year, country, type of publication, primary or secondary research, qualitative or quantitative research); (b) design of quantitative studies (prospective or retrospective observational, case control, quasi-experimental, case studies, organization in health systems and services); (c) methodologies, models or theoretical frameworks used; (d) health context (e) population sample; (f) pharmacist interventions (PIs) performed; (g) results measured; (h) barriers and facilitators revealed; and (i) findings related to the integration of the holistic approach in the patient care process. As this was a scoping review, no restrictions were applied based on the design of the studies or the specifics of the obtained results. Because of the heterogeneity of the selected studies, a formal quality assessment was not feasible [33,34,35].

### 2.3. Reporting and Analysis of the Results

Through tabulation, the studies included in the review were characterized quantitatively with frequencies and percentages. The barriers and facilitators outlined by the authors in the references, findings of the qualitative studies and key conclusions of secondary research were summarized in tabular text form [37].

To probe the presence of holistic approach in the provision of the CMM service, we examined the ways in which different aspects of the patient’s life contexts were explicitly taken into account. We focused mainly on three aspects: (1) the identification of demographic and psychosocial variables that are associated with patient eligibility and the presence of diseases or DRPs; (2) the usefulness of psychosocial aspects and patient’s medication experiences in the adaptation and reconciliation of PIs; and (3) the results of PIs from the patient’s perspective [20,21,38].

## 3. Results

**Figure 1:**
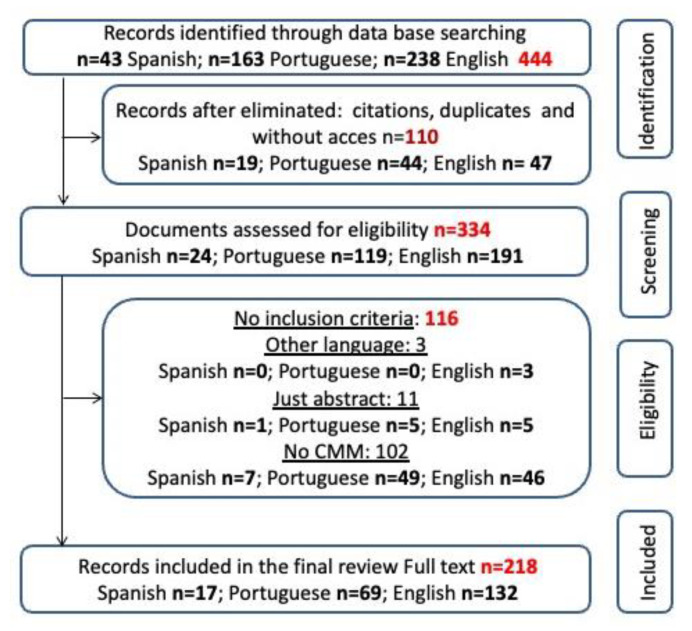
Flowchart of study selection and inclusion process.

The literature review process is illustrated in Figure 1. A total of 444 documents were identified in the two searches of Google Scholar, resulting in 43 Spanish, 163 Portuguese and 238 English documents. Duplicates (n = 72), citations (n = 28) and documents that could not be accessed (n = 15) were eliminated. Of the remaining 334 publications, 218 were retained for further analysis. 

### 3.1. Publication Categorization

The literature search yielded a very diverse set of publications. Of the 218 publications included from 2010 to 2020, the highest number originated in the United States (n = 104; 48%), followed by Brazil (n = 76; 35%) and Spain (n = 11; 5%). There was a preponderance of journal articles (n = 159; 73%) and of these, a small majority were original papers with the primary research (n = 83/159; 52%). Gray literature (59/218; 27%) was mostly represented by master’s theses (n = 23/59; 39%) and doctoral theses (n = 13/59; 22%). In primary research, although quantitative research predominated (n = 117/218; 54%), there was a notable presence of qualitative research (n = 36/218; 16%) mostly in Brazil (n = 22/36; 61%). The techniques applied were interviews (n = 31/36; 86%), participant observations (n = 17/36; 47%), focus groups (n = 3/36; 8%), documentary analysis (n = 4/36; 11%) and one photovoice study. In the category of secondary research (59), reviews (n = 27/59; 46%) stood out among others such as papers presented at events or on websites (n = 7/59; 12%), commentaries/newsletters (n = 7/59; 12%), brief communications (n = 6/59; 10%), editorials (n = 6/59; 10%), manual/electronic textbook (n = 2/59; 4%), guide/procedure (n = 2/59; 4%) and an educational program (n = 1/59; 2%).

### 3.2. Design of Quantitative Studies

Complementing the diverse qualitative research approaches noted above, a range of quantitative investigations was present in the literature search. As part of the process of introducing and implementing the CMM service, quantitative research was dominated by prospective longitudinal observational studies (n = 62/117; 53%) over retrospective studies (n = 34/117; 29%). For the evaluation of the impact of pharmacist interventions, comparisons were most frequently made between groups of patients (n = 23; 20%), but within-group comparisons were also present (n = 14; 12%). Case studies were present (n = 10/117; 8.5%) in addition to research related to the development of tools or methodologies to improve CMM service performance (n = 11/117; 9%).

### 3.3. Theoretical Frameworks Used

Regarding the identification of DRP in the framework of the provision of the CMM service, the classifications used in decreasing order of frequency were Cipolle, Strand and Morley’s seven categories of DRPs (53%) [38], potentially inappropriate medication (PIM) in the older population (10.3%) [39], Hepler and Strand’s eight categories of DRP (9.4%) [40], the Granada Consensus (5.6%) [41], Medication Error (4.7%) [42] and the European Pharmaceutical Care Network classification (3.7%) [2]. The most commonly applied methodologies during the development of the CMM service were Pharmacotherapy Workup [38], Beers Criteria [39] and Dáder Method [43].

### 3.4. Health Context and Population

The research studies were mainly conducted in primary healthcare settings (81%) within primary care centers (40%) and community pharmacies (33%). In most quantitative studies, patients over 18 years of age were studied (n = 88/107; 82%), although in some studies where general results are shown, the age ranges are not specified (n = 18/107; 17%). A substantial percentage was only aimed at older adults over 60 (n = 36/107; 34%). The five most prevalent diseases were diabetes mellitus, hypertension, myocardial failure, mental illness and hyperlipemia [44]. Researches (27%) characterized the pharmaceutical service implemented based on the experiences of the different stakeholders involved (pharmacists, physicians, nurses, patients) and mostly used qualitative research methodology (93%).

### 3.5. Interventions Carried out by Pharmacists and Results of Implementation Studies

All articles specify the levels of acceptance and the target population of the pharmacist interventions. PIs are categorized by authors into those that resolve or prevent DRPs, provide patient education and refer patients to other professionals in the healthcare system. PIs are identified by those carried out in the context of pharmacist–patient or pharmacist–prescriber interaction and to a lesser extent for its clinical significance. Among the most frequent actions reported by the authors are the provision of patient education, recommendations for dosage adjustments, initiation, change in or discontinuation of medications and the proposal of patient monitoring with clinical and laboratory parameters. In 32 of the quantitative studies, PI actions were not detailed. PIs were not described in the descriptive and retrospective studies [45,46,47,48,49,50,51,52,53,54,55,56,57,58,59,60]. Other sources only focused on the impacts or level of acceptance of PIs or just show general results of the service [61,62,63,64,65,66,67,68,69,70,71,72,73,74,75,76].

Different results were identified as measures of the provision of the CMM service, which are listed in Table 1. Two or more results were measured in 75% of the quantitative studies.

There were obstacles (Table 2) and facilitators (Table 3) that affected or contributed to improving the CMM service development, according to the authors. 

### 3.6. Holistic Approach in the Provision of CMM Service

As documented in the introduction, we also aimed to estimate the prevalence of non-medication-related aspect in the research about CMM. A minority of studies reported on a holistic approach, 15% (n = 16) in quantitative research and 19% (n = 7) in qualitative research. This approach was evidenced by the elicitation of patient’s medication experiences associated with drug-related problems, diseases or the provision of services [90,106,131,132,133,134,135,136,137]. As part of the pharmaceutical care process or as criteria for the provision of CMM services, demographic data, psychosocial variables and physical and psychological quality of life scales were analysed [56,89,107,109,112,128,138,139,140]. Some authors specify that PIs were tailored based on the social context of the patient, for example, the schedules and habits of daily life, exercises, diet, and smoking habits [14,108,127]. Patients’ meanings, beliefs, concerns, and interpretations of medication and illness have been considered and reconciled in PIs [132,141,142].

## 4. Discussion

### 4.1. Publication Characteristics and Their Main Contributions

In this study, we reviewed a large number of publications written in three languages that were using dissimilar methodologies. We can gain a better understanding of the implementation of CMM services globally by examining the different investigations listed in this scoping review.

The United States (USA) is prominently represented because CMM services have been implemented in many, if not all, of the states [143]. There is already experience in this country with pharmacists using SDOH data [144,145].

In Spain, interesting clinical cases have been published with a holistic approach [14,108,146]. In this country, a guide was published to implement the CMM service [147].

In Latin America, CMM service is accepted in countries such as Cuba, Argentina and Colombia [5,45,148,149], though Brazil clearly stands out with its implementation of CMM services (Gerenciamento da Terapia Medicamentosa). In recent years, several studies demonstrated its clinical impact on patients, particularly those conducted in primary care and among chronic disease patients [70,84,86,124,150,151,152,153,154,155]. Various qualitative research methods and techniques were applied in this research, such as phenomenology [134,135,156], ethnography [80,157,158,159], autoethnography [160,161,162,163,164,165], action research [166], grounded theory [167]; interview [104,168], participant observation [169,170], focus group [19], photovoice [133] and analysis of documents [171]. The majority of results originating from Brazil were published in Portuguese or Spanish explaining why these insights about CMM services are not more widely known yet.

To characterize the CMM implementation, it was important to include the gray literature in the review. Specifically, the thesis work (master’s, doctoral, bachelor’s and residency) demonstrated the connection between pharmacy and academic training, providing a platform for the implementation, continuity and improvement of CMM services [47,50,61,81,90,91,99,100,104,110,113,123,127,134,135,156,157,158,160,161,167,172,173,174,175,176,177,178,179,180,181,182]. Research from Brazil confirms that the implementation of the CMM service has been achieved through research projects, strengthening the connection between the academy and the health system [183]. The USA authors report that students improved the completion rate of medication reviews and increased PI productivity, thereby generating additional revenue [184]. 

The majority of the review studies summarize clinical, humanistic, and economic outcomes in different health contexts and demonstrate the impact of PIs onpatients [180,185,186,187,188,189]. In other reviews, the challenges and limitations of clinical practice are examined, as well as the benefits of electronic records and different modes of communication with patients (face-to-face, telephone, video) [94,117,143,190,191,192]. A wide range of publications discuss CMM services and pharmacists’ role in their implementation, such as articles on websites, commentaries, brief communications, guides, and editorials, among others [117,118,185,193,194,195,196,197,198,199,200,201,202,203,204,205,206,207,208]. There are reports about the transition from one level of healthcare to another; patients and healthcare institutions have benefited from clinical pharmacist services [115,118,209,210]. A number of sources offer new suggestions for improving CMM service provision through guidelines, models or tools [98,99,174,193,198,211,212,213,214,215,216,217,218]. While those proposals may constitute important references for future implementations of the CMM service, we recommend that pharmaceutical care be delivered with more efficient tools tailored to the patient’s context.

In contrast to Brant’s research [28], qualitative studies did not numerically dominate our scoping review. Some studies included aspects related to pharmacist attitudes [219,220]. Additionally, pharmacists are recognized by healthcare team members for understanding patient needs and taking responsibility for patient outcomes [221]. Furthermore, pharmacist integration has been linked to clinical benefits for patients, time savings and improved workflows [222]. Pharmacists need to provide customized solutions to individual patient problems, as well as provide patient education and ensure their satisfaction [223,224].

The qualitative research with a holistic vision of the DRPs allowed understanding of the DRPs from the patient perspective. Through the analysis of patients’ discourse, insight was gained into the patient needs associated with pharmacotherapy and the complexities of family and social environments. It was possible to explore factors influencing non-adherence such as lack of understanding of instructions or language, the occurrence of adverse reactions, or simply concern about the risks associated with pharmacotherapy [133,166].

### 4.2. Holistic Approach to the Implementation Process

It proved useful to analyze the holistic dimensions of various medication-related services, such as CMM, described in the literature. The prevalence of the Pharmacotherapy Workup [38] in the theoretical framework of the CMM service might be due to its ability to provide a comprehensive description and evaluation of the patient. Pharmacotherapy Workup proposed that open-ended questions are useful for obtaining subjective experiences on the medication and the disease [38]. The current review noted that medication review may overlap with the provision of CMM service [64,87,110,225,226]. In essence, however, the medication review service [227] needs to be more comprehensive in nature and record other patient details [228].

The results demonstrate the potential benefits received by patients at all levels of healthcare. In this regard, Kuo et al. commented on CMM’s versatility and promising impact on healthcare quality across multiple settings [229]. Most implementations were oriented to people with chronic diseases and the elderly, but in addition we note the studies on prisoner populations [137] and transgender individuals [134], illustrating McFarland’s statement on the nearly universal applicability of CMM [230].

The effect size of PIs as impact criteria on clinical parameters, health problems, cost reduction and increase in profits is a measure of the direct impact the pharmacist had on patient health and quality of care system [68,69,75,175,231,232]. However, non-pharmacological treatment, care received from other health professionals, the placebo effect and remission of disease can all contribute to the results.

In this review, published studies generally show that pharmacists were focused on achieving clinical and economic outcomes and the resolution or prevention of DRPs [70,71,72,73,74,75,76]. It is necessary to clarify the ways to reach those outcomes without losing sight of the impact on patient satisfaction. A recent review study, assessing the Impact of CMM on achieving the Quadruple Aim, reported high levels of patient satisfaction [230].

In addition to the PIs that modify the pharmacotherapy, patient counseling by pharmacists contributes to improved well-being and the quality of life [233]. However, patient perspectives were only sporadically incorporated into health outcomes [47,61] and as discussed by Stewart et al., very little is known about patients’ perspectives on the effectiveness of medicine consultations [29].

There are studies that analyze psychosocial variables in relation to health problems, medication or DRPs [57,90,110,113,138,139,140], but the usefulness of SDOH in PIs and the impact they may have on patient outcomes is not fully known yet. The American Society of Hospital Pharmacy already stated that across the continuum of care, pharmacists must ensure that determinants of health are integrated into overall approaches to individual patient and population health interventions [234]. Oliveira et al. point out that the cultural diversity and educational level should be considered [235,236]. Social workers should be included in healthcare teams; their interaction improves the health and quality of life of patients, according to Rust and Davis [237]. Recently, a conceptual model was proposed that incorporates SDOH in the patient care process of identification, prevention and resolution of DRPs [20]. 

There are a small number of studies that gauge the patient’s medication experiences through open-ended questions [91,107,131,132,133,134,135,136,137]. Oliveira et al. suggest balancing the objective and subjective elements of patient care process in CMM services [21]. A pharmacist can use the patient’s medication experiences to assist them in overcoming their barriers and adapting their pharmacotherapy as needed. Medication experience is supportive evidence for identifying DRPs [105,238].

In relation to the practice centered on the patient, Dolovich states that it will be valuable for pharmacy to refine study designs and outcome measures that can better quantify the effect of the pharmacist utilizing the holistic approach [239]. Therefore, quantitative measurement tools should be designed that take into account the different dimensions revealed in the qualitative studies.

Finally, despite the progress achieved, most of the referents do not yet reflect a broader view of the patient’s life situation and its relationship to pharmacotherapy and the ways in which the pharmacist implements holistic elements to solve or prevent DRPs.

### 4.3. Study Limitations

This scoping review has some limitations. Our search was limited to research published since 2010, as we prefer to focus on recent results due to the constantly evolving nature of CMM [7]. Languages other than Spanish, Portuguese and English were not included in the search and this may have precluded us from describing efforts in other parts of the globe such as the Arabic world of the Far East. The search was limited to Google Scholar, and although some papers may have been missed, this was counterbalanced by its efficiency in providing the gray literature. Another limitation is the inherent difficulty of synthesizing methodologically diverse studies and of assessing their quality, making it challenging to account for the relative importance of the individual publications.

## 5. Conclusions

In the conducted review, the impacts achieved with the implementation of the CMM service in the clinical, humanistic and economic order in patients and in the quality of health care were verified. However, this scoping review confirmed the limited implementation of the holistic approach in CMM services. Few studies have examined the effect of SDOH and the patient’s medication experiences in the patient care process. Work is therefore needed on the ways in which and whether a pharmacist implements holistic elements in their practice to resolve DRPs. The literature review additionally reveals the persistence of numerous and diverse barriers that continue to limit the provision of comprehensive and holistic pharmaceutical care to patients. In this study, a large number of publications of different types were reviewed and the search did not focus on a particular healthcare setting or specific medical conditions of the patient. Therefore, our results are useful for providing an overview of the implementation of the CMM service and the holistic approach in particular.

## Figures and Tables

**Table 1:** Results reported in the quantitative studies about CMM.

Results	Studies n = 107 (%)
1. Drug-related problems identified, resolved or prevented	31 (28.9)
2. Monitoring of clinical parameters and laboratory tests	27 (25.2)
3. Reduction in costs	25 (23.3)
4. Clinical progress and resolution of health problems	18 (16.8)
5. Level of acceptance of pharmacist intervention	24 (22.4)
6. Increased therapeutic adherence	10 (9.3)
7. Decreased use of health services	12 (11.2)
8. Number of medications with potentially inappropriate indications	10 (9.3)
9. Patient satisfaction	9 (8.4)
10. Reduction in the number of DRPs	6 (5.6)
11. Deprescription or decrease in drug consumption	5 (4.6)
12. Improvement in quality of life	5 (4.6)
13. Reduction in the number of adverse reactions	3 (2.8)
14. Improvement in quality of prescription parameters	3 (2.8)
15. Increased medication knowledge among patients	2 (1.8)
16. Favorable patient medication experience	2 (1.8)

**Table 2:** Barriers reported in the studies about CMM.

Structure	Studies’ References
1. Insufficient time to provide patient care	[68,73,77,78,79,80]
2. Need for education or training	[61,70,78,81]
3. Space requirement for the service	[77,78]
4. Lack of material resources	[82]
5. Service documentation needs to be standardized	[82]
6. Pharmacy non-payment	[78,82]
7. Difficulties in curricular training	[78]
**Patient Care Process**	
1. Interprofessional collaboration difficulties	[72,83,84,85,86]
2. Lack of access to medical records	[87]
3. Recruitment and eligibility difficulties prevented patients from benefiting	[58,88]
4. Influence of the communication modalities on the acceptance or performance of Pharmacist Interventions	[56,65]
5. Problems of accessibility to medication	[48,53,85,88,89,90,91,92]
6. Poor quality of patient documentation	[93,94]
7. Loss of patients to follow-up	[84,91,95,96,97]

**Table 3:** Facilitators identified in the studies about CMM.

Structure	Studies’ References
1. Link with undergraduate and graduate students	[64,79,97,98,99]
2. Model or tool to guide the provision of the service	[100,101,102]
3. The various activities of the pharmacy are carried out in addition to the provision of clinical services to the patient	[63,103,104]
4. Remuneration to the pharmacist (contractual models or programs)	[63,95,105]
5. Service as a source of learning and training	[106]
**Patient Care Process**	
1. Electronic records that facilitate the activity	[107]
2. More holistic description of the patient	[14,84,106,108,109]
3. Incorporation of the pharmacist as member of the health team	[63,68,84,90,92,98,105,108,110,111,112,113]
4. Experiences of community pharmacy–hospital collaboration	[103,112,114,115,116]
5. Usefulness of virtual patient follow-up	[95,117,118,119]
6. Trustful pharmacist–patient relationship facilitates communication	[62,72,74,86,97,98,120]
7. Cost reduction on pharmacy after PIs	[71,84,105,111,119,121,122,123,124,125,126]
8. Assessment of clinical parameter status after PIs	[115]
9. Assessment of quality of life or disease progression	[14,88,124]
10. Achieving patient satisfaction through service delivery	[66,105,120,127]
11. Assessment of patients’ medication experiences and self-perceptions of health	[58,128,129,130]

PIs: pharmacist interventions.

## Data Availability

Not applicable.
